# The effect of Kenya’s free maternal health care policy on the utilization of health facility delivery services and maternal and neonatal mortality in public health facilities

**DOI:** 10.1186/s12884-018-1708-2

**Published:** 2018-03-27

**Authors:** C. M. Gitobu, P. B. Gichangi, W. O. Mwanda

**Affiliations:** 1 Institute of Tropical and Infectious Diseases (UNITID), University of Nairobi, Nairobi, Kenya; 2 Department of Human Anatomy, University of Nairobi, Nairobi, Kenya; 3 Department of Obstetrics and Gynecology, University of Ghent, Ghent, Belgium; 4 Department of Human Pathology, University of Nairobi, Nairobi, Kenya

**Keywords:** Free maternal health care policy, Maternal mortality ratio, Neonatal mortality rate and health facility delivery services utilization

## Abstract

**Background:**

Kenya abolished delivery fees in all public health facilities through a presidential directive effective on June 1, 2013 with an aim of promoting health facility delivery service utilization and reducing pregnancy-related mortality in the country. This paper aims to provide a brief overview of this policy’s effect on health facility delivery service utilization and maternal mortality ratio and neonatal mortality rate in Kenyan public health facilities.

**Methods:**

A time series analysis was conducted on health facility delivery services utilization, maternal and neonatal mortality 2 years before and after the policy intervention in 77 health facilities across 14 counties in Kenya.

**Results:**

A statistically significant increase in the number of facility-based deliveries was identified with no significant changes in the ratio of maternal mortality and the rate of neonatal mortality.

**Conclusion:**

The findings suggest that cost is a deterrent to health facility delivery service utilization in Kenya and thus free delivery services are an important strategy to promote utilization of health facility delivery services; however, there is a need to simultaneously address other factors that contribute to pregnancy-related and neonatal deaths.

**Electronic supplementary material:**

The online version of this article (10.1186/s12884-018-1708-2) contains supplementary material, which is available to authorized users.

## Background

The reduction and elimination of pregnancy-related mortality remain a challenge in most low income countries [1]. The maternal mortality ratio and the neonatal mortality rate in Kenya have been found to be 362/100,000 live births and 22/1000 live births, respectively [2]. Given that only 61.2% of deliveries in the country are conducted in health facilities, pregnancy-related deaths have been attributed to delivery without skilled birth attendance [2]. Globally, high quality health facility delivery services have been recommended as a solution to preventable maternal and neonatal deaths [3]. For this reason, many African countries have either reduced or eliminated delivery fees to promote health facility delivery service utilization [4].

Kenya joined other African countries in the abolishment of delivery fees in all public health facilities through a presidential directive signed into effect on June 1, 2013 [5]. Through this policy, public health facilities are reimbursed for costs incurred while providing delivery services through a capitation fund provided by the Ministry of Health. This policy provides equal reimbursement for both spontaneous vaginal deliveries and caesarean sections. The amounts reimbursed to health facilities are based on their capacity to manage pregnancy and delivery complications. As such, 2500 Kenya shillings (25 US dollars) are reimbursed for every delivery conducted in level 2 facilities (health centers) and level 3 health facilities (sub district hospitals); 5000 Kenya shillings (50 US dollars) are reimbursed for every delivery carried out in level 4 health facilities (district hospitals) and level 5 health facilities (provincial hospitals); and 17,500 Kenya shillings (175 US dollars) are reimbursed for every delivery performed in national referral health facilities [6].

While eliminating delivery fees is a commendable intervention, pregnancy-related mortality due to the following “three delays” remains a concern: delays in deciding to seek skilled delivery services, delays in arriving at health facilities and delays in receiving adequate treatment and referral [7]. Cost is not the only factor hindering the utilization of health facility delivery services. In Kenya, maternal and neonatal deaths have been attributed to other factors, including lack of transport, long distances to health centers, poorly equipped health facilities, low quality of care in health facilities and traditional and cultural practices [8, 9]. Therefore, while elimination of delivery fees in Kenyan public health facilities partially addresses economic barriers to maternal health care utilization, other economic barriers, health system gaps, quality of health facility delivery services and political, social, environmental and religious factors that may influence the utilization and outcomes of maternal health care in the country have not been addressed [10–15].

In addition, initial assessments of the implementation of this policy have identified various gaps, such as drug and supply shortages, insufficient funding, skilled health care worker shortages, lack of skills among health workers, stakeholder non-involvement in the policy design, delayed reimbursement of costs incurred while providing free maternal health care, heavy workloads, health worker demotivation, healthcare worker attitudes, low privacy levels in public health facilities and unavailability of ambulances for emergencies occurring at community level [16, 17]. In light of these contextual gaps, this study aimed to investigate the effects of the free maternal health care policy in Kenya on health facility delivery service utilization and maternal mortality ratio and neonatal mortality rate in public health facilities.

## Methods

A time series analysis was performed with the period of interest being 24 months before policy implementation (June 1, 2011, to May 31, 2013) and 24 months after policy implementation (June 1, 2013, to May 31, 2015).

The study was conducted in 77 public health facilities selected from 14 counties in the Republic of Kenya. At the time of data collection, Kenya’s public health care facilities were organized in a hierarchical pyramidal structure comprising six levels, namely, level 1 health facilities (health centers), level 2 health facilities (dispensaries), level 3 health facilities (sub district hospitals), level 4 health facilities (district hospitals), level 5 health facilities (provincial hospitals) and level 6 health facilities, which were national referral hospitals [18]. Caesarean sections are carried out in level 4, 5 and 6 health facilities and therefore, health facilities in these three levels were the study sites. This hierarchical pyramidal structure is expected to change from six to four tiers once relevant legislation is passed by the national parliament [19–22].

Deceased mothers and deceased neonates from the selected health facilities were included in the assessment of maternal and neonatal mortality. Mothers who had delivered in the selected health facilities during the 4 years under consideration were included in the assessment of health service utilization.

Fourteen of the forty-seven counties in the Republic of Kenya were selected for inclusion in the study after single-stage cluster sampling and subsequent simple random sampling procedures were applied [23]. The 47 counties were classified into high risk, medium risk and low risk maternal mortality categories based on their perennial maternal mortality ratios. Of these counties, five with a high risk (maternal death to females population ratio above 0.00018), five with a medium risk (maternal death to females population ratio between 0.00012 and 0.000183) and four with a low of risk maternal mortality (maternal death to females population ratio below 0.00012) were included in the study; these studies were selected via simple random sampling. Of the 97 health facilities eligible for inclusion in the study, 77 in 14 counties were selected through stratified multi-stage sampling with the maternal mortality risk, counties, status of health facilities and location being the strata [18]. These health facilities were one maternity nursing home (equivalent of a level 5 health facility in terms of infrastructure and human resource), 58 level 4 health facilities, 17 level 5 health facilities and one level 6 public health facility.

Written consent was obtained from heads of health facilities included in the study as well as from the Director of medical services, Ministry of Health Kenya. Health facilities were assigned unique identification codes which are only known to the authors.

The authors relied on primary data sources in every health facility (maternity ward registers, audited records of maternal deaths, neonatal ward registers and death registers) as opposed to DHIS records to ensure data accuracy in the study. The instruments for data collection in this study were tabulated questionnaire to capture monthly figures on neonatal mortality rate, maternal mortality ratio and health facility delivery services utilization in each of the 77 health facilities. The data collection instruments were pre tested at a level 4 health facility. Four research assistants, we recruited, trained and used in data collection.

The instrument used for data collection was a tabulated questionnaire designed to capture monthly neonatal mortality rates, maternal mortality ratios and health facility delivery service utilization data for each of the 77 health facilities. SPSS (IBM version 23) was used for data analysis, and the results were stratified by geographical location and health facility level. Interrupted time series analyses of quarterly (3-month) maternal mortality ratios, neonatal mortality rates and health facility delivery service numbers were performed using autoregressive integrated moving average (ARIMA) models, and the level of significance was set at *p* <  0.05. Diagnostic tests were performed to assess the general fit of the model, and stationary R-squared and traditional R-squared (R^2^) values were calculated. Root Mean Square Error (RMSE) which is the standard deviation of the residuals (prediction errors) was used to measure the spread out of residuals. Lastly, Ljung-Box statistic, which is a function of the accumulated sample autocorrelations, was used as a diagnostic tool to test the lack of fit of a time series model through autocorrelations of the residuals.

## Results

### Health facility delivery service utilization

A statistically significant increase in the number of deliveries in the health facilities was identified; this number increased from 234,601 before policy implementation to 303,705 after policy implementation, representing a 29.5% increase (*p* <  0.05; Table 1).

**Table 1:** Total Deliveries in the different levels of health facilities

Variable	Variable Description	Total Deliveries Pre- Policy	Total Deliveries Post-Policy	*P* Value
Location	Rural-Based facilities	88,153.00	112,321.00	< 0.001
	Urban-Based facilities	146,448.00	191,384.00	< 0.01
Facility Level	Maternity Nursing Home	39,729.00	43,411.00	< 0.05
	Level 4 facilities	113,950.00	159,956.00	< 0.001
	Level 5 facilities	60,303.00	74,646.00	0.06
	Level 6 facility	20,619.00	25,692.00	0.10
All 77 facilities		234,601.00	303,705.00	< 0.001

The results of the analysis of quarterly deliveries in the 77 health facilities indicated a decreasing trend in deliveries (slope = − 13.131, *p* = 0.00) during the 24 months preceding implementation of the policy. Thus during the 24 months before the intervention, no significant change was identified in the number of facility-based deliveries. A significant increase, however, in the quarterly number of facility-based deliveries (slope = 124.90, *p* <  0.01) in the 77 health facilities was identified after policy implementation (Table 2).

**Table 2:** Quarterly Patterns in Health Facility Delivery Service Utilization

	Slope 24 months pre-policy	Slope 12 months pre-policy	Slope 6 months pre-policy	Slope 3 months pre-policy	Slope 3 months post-policy	Slope 6 months post- policy	Slope 12 months post-policy	Slope 24 months post-policy
All 77 facilities	−13.13 (*p* = 0.63)	−13.13 (*p* = 0.63)	− 124.90 (*p* < 0.05)	− 13.13 (*p* = 0.063)	111.77 (*p* < 0.05)	111.77 (*p* < 0.05)	− 26.65 (*p* = 0.18)	124.90 (*p* < 0.05)
Urban-based facilities	− 17.37 (*p* = 0.46)	− 17.37 (*p* = 0.46)	−77.10 (*p* < 0.05)	−17.37 (*p* = 0.46)	59.74 (*p* < 0.05)	59.74 (*p* < 0.05)	111.77 (*p* < 0.05)	77.10 (*p* < 0.05)
Rural-based facilities	4.65 (*p* = 0.44)	4.65 (*p* = 0.44)	−47.34 (*p* < 0.05)	4.65 (*p* = 0.44)	51.99 (*p* < 0.05)	51.99 (*p* < 0.05)	59.74 (*p* < 0.05)	47.34 (*p* < 0.05)
Maternity hospital	−3.12 (*p* = 0.87)	−3.12 (*p* = 0.85)	23.54 (0.45)	−3.12 (*p* = 0.87)	−26.66 (*p* < 0.052)	− 26.66 (*p* < 0.05)	51.99 (*p* = 0.0)	− 23.54 (*p* = 0.45)
Level 4 facilities	18.79 (*p* = 0.15)	18.79 (*p* = 0.15)	−68.00 (*p* < 0.05)	18.79 (*p* = 0.15)	86.79 (*p* < 0.05)	86.789 (*p* < 0.05)	86.98 (*p* < 0.05)	68.00 (*p* < 0.05)
Level 5 facilities	−19.59 (0.13)	19.59 (*p* = 0.13)	−53.63 (*p* < 0.05)	− 19.59 (*p* = 0.13)	34.04 (*p* < 0.05)	34.04 (*p* < 0.05)	34.04 (*p* < 0.05)	53.63 (*p* < 0.05)
Level 6 facility	−0.75 (*p* = 0.87)	− 0.76 (*p* = 0.09)	− 0.76 (*p* < 0.05)	16.26 (*p* < 0.05)	16.26 (*p* < 0.05)	16.26 (*p* < 0.05)	16.26 (*p* < 0.05)	17.01 (*p* < 0.05)

A closer look at the delivery service utilization trends identified during the 6 months before policy implementation indicates the presence of a decreasing trend in the utilization of facility-based delivery services (slope = − 124.90, *p* = 0.02). During the 6 months after policy implementation, this trend reversed, and a significant increase in the number of deliveries in health facilities was observed (slope = 111.77, *p* <  0.001).

Both the stationary and the traditional R^2^ tests yielded a value of 0.73, implying that 73% of the model was explained by the policy intervention. In addition, a root mean square error (RSME) value of 384.22 was identified, suggesting that a large portion of the variability observed in the number of deliveries could be explained by the predictive model. The mean absolute percentage error (MAPE) value of 7.25 indicated that the values predicted using the policy implementation model were, on average, within 7.25% of the actual values (Additional file 1).

Both the stationary R-squared and traditional R-squared values for all 77 health facilities were 81.5% (*P* = 0.15; Additional file 2). The stationary R-squared and traditional R-squared values varied from 73.1% to 43.7% across various categories of health facilities. This finding indicated that although policy implementation resulted in a remarkably higher number of facility-based deliveries, this intervention had a non-uniform effect on delivery service utilization across the 77 health facilities (*p* = 0.15).

### Maternal mortality ratio

A nonsignificant decrease in the ratio of maternal mortality in the 77 health facilities was identified, with the mortality ratio decreasing from 258.3/100,000 live births to 237.1/100,000 live births (*p* = 0.07) following policy implementation (Table 3). It is only in the rural areas that a significant decline in maternal mortality ratio was recorded.

**Table 3:** Maternal Mortality Ratios

Variable	Variable Description	MMR Pre-Policy	MMR Post-Policy	*P* Value
Location	Rural-Based facilities	158.20	116.70	0.02
	Urban-Based facilities	326.00	324.40	0.83
Facility Level	Maternity Home	44.80	43.10	0.52
	Level 4 facilities	181.50	182.60	0.19
	Level 5 facilities	254.10	196.70	0.11
	Level 6 facility	1125.80	983.30	0.48
All 77 facilities		258.30	237.10	0.07

The ARIMA model parameters for the pre-intervention slope that was calculated using data from the 77 health facilities showed a nonsignificant decrease in the rate of quarterly maternal mortality (slope = − 1.64, *p* = 0. 20) during the 24 months preceding user fee removal. During the 24 months after free maternity health care services were first offered, a significant increase in the rate of quarterly maternal mortality was observed in the health facilities under consideration (slope = 3.49, *p* = 0.01). This finding indicated that the free maternal health care policy did not have a significant effect on facility-based maternal mortality ratios (Table 4).

**Table 4:** Quarterly Trends in Maternal Mortality Ratio

Facility	Slope 24 months pre-policy	Slope 12 months pre- policy	Slope 6 months pre-policy	Slope 3 months pre-policy	Slope 3 months post-policy	Slope 6 months post-policy	Slope 12 months post-policy	Slope 24 months post-policy
All 77 health facilities	−1.64 (*p* = 0.20)	−1.64 (*p* = 0.20)	−5.12 (*p* < 0.05)	− 1.64 (*p* = 0.20)	3.486 (*p* < 0.05)	3.49 (*p* < 0.05)	3.48 (*p* < 0.05)	3.47 (*p* < 0.05)
Urban-based facilities	− 1.92 (*p* = 0.51)	− 1.92 (*p* = 0.51)	−7.00 (*p* = 0.09)	− 1.92 (*p* = 0.51)	5.09 (*p* = 0.08)	5.09 (*p* = 0.08)	5.09 (*p* = 0.08)	5.09 (*p* = 0.08)
Rural-based facilities	− 1.38 (*p* = 0.40)	− 1.38 (*p* = 0.40)	− 2.37 (*p* = 0.31)	− 1.38 (*p* = 0.40)	0.99 (*p* = 0.54)	0.99 (*p* = 0.54)	0.99 (*p* = 0.54)	0.99 (*p* = 0.54)
Maternity hospital	− 0.08 (*p* = 0.94)	− 0.08 (*p* = 0.94)	− 2.25 (*p* = 0.17)	− 0.08 (*p* = 0.94)	2.17 (*p* = 0.06)	2.17 (*p* = 0.06)	2.17 (*p* = 0.06)	2.17 (*p* = 0.06)
Level 4 facilities	−2.61 (*p* = 0.20)	− 2.61 (*p* = 0.20)	−6.85 (*p* < 0.05)	− 2.61 (*p* = 0.20)	4.24 (*p* < 0.05)	4.24 (*p* < 0.05)	4.24 (*p* < 0.05)	4.24 (*p* < 0.05)
Level 5 facilities	−4.51 (*p* = 0.06)	− 4.51 (*p* = 0.06)	− 6.70 (*p* = 0.05)	− 4.51 (*p* = 0.06)	2.19 (*p* = 0.36)	2.19 (*p* = 0.36)	2.19 (*p* = 0.36)	2.19 (*p* = 0.36)
Level 6 facility	8.50 (*p* = 0.42)	8.50 (*p* = 0.42)	11.05 (*p* = 0.46)	8.50 (*p* = 0.42)	− 2.55 (*p* = 0.81)	− 2.55 (*p* = 0.81)	− 2.55 (*p* = 0.81)	− 2.55 (*p* = 0.81)

Both the stationery R-squared and traditional R-squared (R^2^) values for the model were 0.126, implying that only 12.6% of the variance observed in maternal mortality ratio could be explained by the free maternal health care policy intervention. The RSME value of 112.67 indicated that the interrupted time series model was reliable in predicting maternal mortality trends. The calculated MAPE indicated a 33.77% variation from the model prediction following the policy intervention (Additional file 3).

Overall, the Ljung-Box test statistics were not significant for the 77 health facilities (*p* = 0.54). Significant Ljung-Box test statistics were identified for level 5 health facilities only (30.64, *p* <  0.05). The stationary R-squared and traditional R-squared values indicated that only a minimal decline in maternal mortality ratio occurred in the 77 health facilities (0.19), with the greatest decline in maternal mortality ratio identified in the level 6 health facility (0.56; Additional file 4). The maternal mortality ratios demonstrated a decreasing trend in the health facilities but exhibited marked although not consistently uniform seasonality. Thus, the free maternal health care policy intervention had a random and nonsignificant effect on maternal mortality ratios across all health facilities.

### Neonatal mortality rate

A nonsignificant decline in neonatal mortality rates was identified, with rates decreasing from 23.3/1000 live births to 22.9/1000 live births (*p* = 0.14) following policy implementation (Table 5).

**Table 5:** Neonatal Mortality Rates

Variable	Variable Description	NMR Before Policy Implementation	NMR After Policy Implementation	*P* Value
Location	Rural-Based facilities	10.30	9.90	0.21
	Urban-Based facilities	35.10	34.20	0.45
Facility Level	Maternity Hospital	24.20	24.90	0.51
	Level 4 facilities	7.30	6.60	0.17
	Level 5 facilities	26.90	26.40	0.81
	Level 6 facility	102.30	104.40	0.54
All 77 facilities		23.30	22.90	0.14

The pre-intervention slope indicated a nonsignificant decreasing trend in the quarterly neonatal mortality rates across all health facilities over the course of the 24 months preceding policy implementation (slope = − 0.09, *p* = 0.24). Implementation of the policy did not significantly affect neonatal mortality rates during the first 24 months following policy implementation (slope = 0.12, *p* = 0.10; Table 6).

**Table 6:** Quarterly Trends in Neonatal Mortality Rate

Facility	Slope 24 months pre-policy	Slope 12 months pre-policy	Slope 6 months pre-policy	Slope 3 months pre-policy	Slope 3 months post-policy	Slope 6 months post- policy	Slope 12 months post- policy	Slope 24 months post-policy
All 77 health facilities	− 0.09 (*p* = 0.24)	− 0.09 (*p* = 0.24)	− 0.21 (*p* = 0.05)	− 0.09 (*p* = 0.24)	0.12 (*p* = 0.10)	0.12 (*p* = 0.10)	0.12 (*p* = 0.10)	0.12 (*p* = 0.10)
Urban-based facilities	− 0.10 (*p* = 0.48)	− 0.10 (*p* = 0.48)	− 0.41 (*p* < 0.05)	− 0.10 (*p* = 0.10)	0.32 (*p* < 0.05)	0.32 (*p* < 0.05)	0.32 (*p* < 0.05)	0.32 (*p* < 0.05)
Rural-based facilities	− 0.08 (*p* = 0.10)	− 0.08 (*p* = 0.10)	− 0.05 (*p* = 0.49)	− 0.08 (*p* < 0.05)	− 0.04 (*p* = 0.48)	− 0.04 (*p* = 0.48)	− 0.04 (*p* = 0.48)	− 0.04 (*p* = 0.48)
Maternity hospital	− 0.16 (*p* = 0.24)	− 0.16 (*p* = 0.24)	− 0.53 (*p* < 0.05)	− 0.161 (*p* = 0.24)	0.37 (*p* < 0.05)	0.37 (*p* < 0.05)	0.37 (*p* < 0.05)	0.37 (*p* < 0.05)
Level 4 facilities	− 0.09 (*p* = 0.06)	− 0.09 (*p* = 0.06)	− 0.12 (*p* < 0.05)	− 0.09 (*p* = 0.06)	0.09 (*p* = 0.05)	0.09 (*p* = 0.05)	0.09 (*p* = 0.05)	0.09 (*p* = 0.05)
Level 5 facilities	0.17 (*p* = 0.27)	0.17 (*p* = 0.27)	0.39 (*p* = 0.07)	0.17 (*p* = 0.27)	− 0.22 (*p* = 0.14)	− 0.22 (*p* = 0.14)	− 0.22 (*p* = 0.14)	− 0.22 (*p* = 0.14)
Level 6 facility	−0.15 (*p* = 0.83)	− 0.15 (*p* = 0.83)	− 0.64 (*p* = 0.52)	− 0.15 (*p* = 0.83)	0.50 (*p* = 0.48)	0.50 (*p* = 0.48)	0.50 (*p* = 0.48)	0.505 (*p* = 0.48)

Only 32.90% of the minimal and nonsignificant change observed in the neonatal mortality rates could be attributed to policy implementation. The RSME and MAPE values for this model were 688.10 and 17.96, respectively (Additional file 5).

The general fit of the model for all 77 health facilities was not significant (*p* = 0.06). Both the stationary R-squared and traditional R-squared values indicated that only 10.5% of the variation could be explained by the model, implying that policy implementation was associated with only a minimal difference in neonatal mortality rates when compared with baseline figures (Additional file 6).

## Discussion

A statistically significant increase in facility deliveries was observed in Kenya following the implementation of the free maternal health care policy in 2013. This result is similar to observations of the implementation of free maternal health care policies in other African countries [24–27]. Applying user fees for delivery services in health facilities may limit the demand, and thus elimination of user fees may improve access to health facility delivery services. The increase in facility-based deliveries remained consistently high over the 2 years post-policy implementation. This finding is in contrast with other regional studies, in which increased utilization of delivery care services was documented during the initial 3 months following user fee removal [27, 28]. The high utilization of free delivery services over a long period of time in this study creates an opportunity to reduce maternal and neonatal mortality.

Implementation of the free maternal health care policy in Kenyan public health facilities did not have a significant effect on maternal and neonatal mortality. This observation is consistent with results of other local and international studies, which have shown user free health policies to have limited or no effect on maternal and neonatal mortality [28–32].

A significant decline in maternal mortality ratio was noted in the rural based health facilities; the Kenya household health expenditure and utilization survey shows that 66% of the country’s population lives in rural areas and the rural population is more likely to use public health services than the urban residents [33]. The Kenya household health expenditure and utilization survey also showed that those in the poorest quintile were more likely to use public health facilities than those in the richest quintile. Although only 12% of the change in maternal mortality ratio is attributed to the free maternal health care policy in Kenya, the reduction in maternal mortality ratio in rural based health facilities may be attributed to a high utilization of free delivery services in rural areas where the largest population and poorer population reside.

As pregnancy-related deaths may be attributed to delays in deciding to seek health facility delivery services, delays in arriving at health facilities and delays in receiving adequate treatment and referral, the findings of this study emphasize the fact that other factors may contribute to pregnancy-related deaths in Kenyan public health facilities [34]. The service readiness availability mapping in 2013 when the free maternal health care policy was implemented in the country shows that only about 28% of health facilities in the country had essential medicines for handling pregnancy related emergencies with 51% having Oxytocin injectables, 26% having magnesium sulphate injectables and 53% having gentamicin injectables [35]. The unavailability of these essential drugs points to the inability of health facilities to handle pregnancy and child birth related complicates hence no changes in maternal and neonatal mortality.

The adequacy of pre-existing healthcare infrastructure, human resources for health, and supply of medical commodities ought to be addressed before waiver of delivery fees given the high demand of services that comes with fees abolishment [36]. As a signatory to the Abuja Declaration, Kenya committed itself to allocating at least 15% of the national budget to the health sector. However, close to two decades after signing the declaration, government funding for health care has remained consistently below 8% of the national annual budget [37]. The national reproductive health strategy in Kenya notes that there are gaps in funding reproductive health services in Kenya (MOH, 2016) [38]. Loss of revenue due to abolishment of delivery fees may lead to poor quality of services despite the increase in health facility delivery services utilization, this would in turn result in shortages of inputs like drugs and other supplies necessary to avert pregnancy related mortalities and demotivation of health care workers [31].

Delays in utilizing free delivery services may occur because of low levels of autonomy, low awareness of the availability and importance of health facility delivery services and low perception of pregnancy risk factors in pregnant women. In addition, the long distances from health facilities, unavailability and high costs of transport services, poor roads and rugged geography may also hinder accessibility of free delivery services [34]. Previous assessments in Kenyan public health facilities have reported health systems gaps in service delivery [37, 38]. These gaps include drug and supply shortages, inadequate health staff to provide care to a high number of mothers seeking delivery services, health worker demotivation, delayed reimbursement of costs incurred when providing free maternal health care services, apathy related to free delivery services due to privacy concerns, poor referral channels and poor quality of care in general. The interplay between these challenges and pregnancy-related mortality needs to be further analyzed and addressed.

User fees have traditionally been seen as a major source of income for health facilities [39]. Given that the free maternal health care policy reimbursements are provided by the National Hospital Insurance Fund (NHIF) which also reimburses health facilities for providing general health services to NHIF members, this double payment is not only duplicative but also inefficient and poorly understood by county health managers who report that they do not know exactly how the funds from the free maternal health care policy should be utilized [40]. With devolution, several changes have occurred regarding health care financing protocols in the counties [39]. Before devolution of health services, facilities kept user fee revenue in their own bank accounts, but these funds are now deposited at the county bank accounts, as such not all health facilities keep the regular NHIF reimbursements and the free maternal health care policy reimbursements. In view of this, concerns have been raised over diversion of the free maternal health care funds by county governments and this too has a negative implication on the quality of delivery services offered by the counties [41]. It is for this reason that stakeholders in health are advising that reimbursement for offering free maternal health care services should be done through the Health Sector Services Fund (HSSF) channel. They also note that the current level of compensating health facilities for delivering free maternal health care services is challenging given that it favours facilities with richer catchment areas thus reducing the facility revenues in areas serving smaller population groups hence compromised quality of services. Similarly, despite all health facilities conducting deliveries, the amounts reimbursed varies per facility level.

### Limitations

Data used in the analyses presented in this manuscript have several notable limitations:

- Given that reimbursement of costs incurred in delivery services is based on the number of deliveries conducted in each health facility, the accuracy of data prior to the policy may have been poor when compared to the post policy implementation data.
- Although most of the findings from this study are consistent with other local and international studies, generalization of findings to depict the national picture in the implementation of the free maternal health care policy may be questioned given the shortcomings in equal sampling of the various facility levels, religious dynamics, geographical dynamics and other contextual factors affecting health facility delivery services utilization in the country.
- The free maternal health care policy has been implemented in all public health facilities in Kenya. This provided limitations in getting control groups (public health facilities where the policy was not implemented) for comparisons.
- The study has focused on the outcomes of the policy implementation during the first 2 years of the policy intervention. The trends before 2011 and the trajectory of the outcomes after the 2 years of consideration are unknown, and it is impossible to know whether the observed trends continues after the 2 years of policy implementation under consideration.
- This study was based on retrospective data based on maternal death audits over a period of 4 years. They may have been confounded by population dynamics and context-specific factors in maternal audits. Data on the exact timing of seeking medical help by mothers was not available as such some of the maternal deaths may have been as a result of late presentation in health facilities.

## Conclusion

The elimination of user fees for delivery services in Kenya resulted in a significant increase in the number of deliveries conducted in Kenyan public health facilities; this result indicates that cost may be a key deterrent to delivery service utilization. This finding implies that removal of user fees for delivery services may serve as an important strategy to increase health facility delivery service utilization. However, this policy intervention appeared to have no significant effect on maternal and neonatal mortality. This lack of effect indicates that low utilization of health facility delivery services may not be the only factor contributing to pregnancy-related deaths in low income countries such as Kenya; low quality of delivery services in health facilities could be a contributing factor. In addition to eliminating fees to improve health service access, there is a need to simultaneously address other social, economic, political and contextual factors that are known to contribute to pregnancy-related deaths.

## Additional files

Additional file 1: Fitness of health facility delivery services Model. This additional file is derived from an analysis of the mean absolute percentage error (MAPE) on all health facilities’ deliveries. (DOCX 13 kb)

Additional file 2: Model statistics of health facility delivery services generated through Ljung. Box analysis of all health facilities’ deliveries. (DOCX 14 kb)

Additional file 3: Fitness of maternal mortality ratio model. This additional file is derived from an analysis of the mean absolute percentage error (MAPE) of maternal mortality ratio in all the 77 health facilities. (DOCX 13 kb)

Additional file 4: Model statistics of maternal mortality ratio generated through Ljung. Box analysis of maternal mortality ratio in the 77 health deliveries. (DOCX 14 kb)

Additional file 5: Fitness of maternal mortality ratio model. This additional file is derived from an analysis of the mean absolute percentage error (MAPE) of neonatal mortality rate in all the 77 health facilities. (DOCX 13 kb)

Additional file 6: Model statistics of neonatal mortality rate generated through Ljung. Box analysis of neonatal mortality rate in the 77 health deliveries. (DOCX 17 kb)

## Abbreviations

APHRC: Africa Population and Health Research Center
ARIMA: Autoregressive Moving Averages
CS: Caesarean Section
DF: Degrees of Freedom
HSSF: Health Sector Services Fund
IBM: International Business Machines
IDRC: International Development Research Centre
MAPE: Mean Absolute Percentage Error
MBChB: Bachelor of Medicine and Surgery
MD: Doctor of Medicine
MMED: Master of Medicine in Obstetrics and Gynecology
MMR: Maternal Mortality Ratio
NHIF: National Hospital Insurance Fund
NMR: Neonatal Mortality Rate
PhD: Doctor of Philosophy
PMRCPath: Masters in Human Pathology
RMSE: Root-Mean-Square Error
SE: Standard Error
SPSS: Statistical Package for Social Sciences
UNITID: Institute of Tropical and Infectious Diseases
US: United States
WHO: World Health Organization
