# Supplementary material for: The effect of Kenya’s free maternal health care policy on the utilization of health facility delivery services and maternal and neonatal mortality in public health facilities
Source: BMC Pregnancy Childbirth. 2018 Mar 27;18:77. doi: 10.1186/s12884-018-1708-2 (PMC5870237; doi:10.1186/s12884-018-1708-2)
Supplement: Supplementary file 1 — Fitness of health facility delivery services Model. This additional file is derived from an analysis of the mean absolute percentage error (MAPE) on all health facilities’ deliveries. (DOCX 13 kb) [file 12884_2018_1708_MOESM1_ESM.docx]

**Additional File 1: Fit of the Delivery Number Model**

| **Fit statistic** | **Mean** | **SE** | **Minimum** | **Maximum** |
| --- | --- | --- | --- | --- |
| Stationery R-squared | 0.73 | 0.16 | 0.44 | 0.90 |
| R -squared | 0.73 | 0.16 | 0.44 | 0.90 |
| RMSE | 384.22 | 241.59 | 105.14 | 778.47 |
| MAPE | 7.25 | 3.24 | 3.62 | 13.35 |
