# Supplementary material for: The effect of Kenya’s free maternal health care policy on the utilization of health facility delivery services and maternal and neonatal mortality in public health facilities
Source: BMC Pregnancy Childbirth. 2018 Mar 27;18:77. doi: 10.1186/s12884-018-1708-2 (PMC5870237; doi:10.1186/s12884-018-1708-2)
Supplement: Supplementary file 2 — Model statistics of health facility delivery services generated through Ljung. Box analysis of all health facilities’ deliveries. (DOCX 14 kb) [file 12884_2018_1708_MOESM2_ESM.docx]

**Additional File 2: Delivery Number Model Statistics**

|  | **Model statistics** | | | **Ljung-Box** | | |
| --- | --- | --- | --- | --- | --- | --- |
| **Model** | **Number of Predictors** | **Stationery R-squared** | **R-squared** | **Statistics** | **DF** | **Significance** |
| All facilities | 3 | 0.82 | 0.82 | 22.87 | 17 | 0.15 |
| Urban based facilities | 3 | 0.73 | 0.73 | 20.71 | 17 | 0.24 |
| Rural based facilities | 3 | 0.90 | 0.90 | 23.00 | 17 | 0.15 |
| Maternity home | 3 | 0.44 | 0.44 | 10.68 | 17 | 0.87 |
| Level 4 health facilities | 3 | 0.89 | 0.89 | 28.10 | 17 | 0.04 |
| Level 5 health facilities | 3 | 0.62 | 0.62 | 26.92 | 17 | 0.06 |
| Level 6 health facility | 3 | 0.70 | 0.70 | 18.09 | 17 | 0.38 |
