# Supplementary material for: The effect of Kenya’s free maternal health care policy on the utilization of health facility delivery services and maternal and neonatal mortality in public health facilities
Source: BMC Pregnancy Childbirth. 2018 Mar 27;18:77. doi: 10.1186/s12884-018-1708-2 (PMC5870237; doi:10.1186/s12884-018-1708-2)
Supplement: Supplementary file 3 — Fitness of maternal mortality ratio model. This additional file is derived from an analysis of the mean absolute percentage error (MAPE) of maternal mortality ratio in all the 77 health facilities. (DOCX 13 kb) [file 12884_2018_1708_MOESM3_ESM.docx]

**Additional File 3: Fit of the Maternal Mortality Ratio Model**

| **Fit statistic** | **Mean** | **SE** | **Minimum** | **Maximum** |
| --- | --- | --- | --- | --- |
| Stationery R-squared | 0.13 | 0.05 | 0.05 | 0.20 |
| R -squared | 0.13 | 0.05 | 0.05 | 0.20 |
| RMSE | 112.66 | 118.61 | 45.75 | 378.37 |
| MAPE | 33.98 | 11.33 | 16.39 | 46.91 |
