# Supplementary material for: The effect of Kenya’s free maternal health care policy on the utilization of health facility delivery services and maternal and neonatal mortality in public health facilities
Source: BMC Pregnancy Childbirth. 2018 Mar 27;18:77. doi: 10.1186/s12884-018-1708-2 (PMC5870237; doi:10.1186/s12884-018-1708-2)
Supplement: Supplementary file 4 — Model statistics of maternal mortality ratio generated through Ljung. Box analysis of maternal mortality ratio in the 77 health deliveries. (DOCX 14 kb) [file 12884_2018_1708_MOESM4_ESM.docx]

**Additional File 4: Maternal Mortality Model Statistics**

|  | **Model statistics** | | | **Ljung-Box** | | |
| --- | --- | --- | --- | --- | --- | --- |
| **Model** | **Number of Predictors** | **Stationery R-squared** | **R-squared** | **Statistics** | **DF** | **Significance** |
| All 77 facilities | 3 | 0.20 | 0.20 | 15.78 | 17 | 0.54 |
| Urban based facilities | 3 | 0.09 | 0.09 | 12.40 | 17 | 0.78 |
| Rural based facilities | 3 | 0.15 | 0.15 | 16.57 | 17 | 0.48 |
| Maternity home | 3 | 0.12 | 0.12 | 19.27 | 17 | 0.65 |
| Level 4 facilities | 3 | 0.12 | 0.12 | 24.63 | 17 | 0.31 |
| Level 5 facilities | 3 | 0.43 | 0.43 | 30.11 | 17 | 0.03 |
| Level 6 facilities | 3 | 0.56 | 0.56 | 20.64 | 17 | 0.24 |
