# Supplementary material for: The effect of Kenya’s free maternal health care policy on the utilization of health facility delivery services and maternal and neonatal mortality in public health facilities
Source: BMC Pregnancy Childbirth. 2018 Mar 27;18:77. doi: 10.1186/s12884-018-1708-2 (PMC5870237; doi:10.1186/s12884-018-1708-2)
Supplement: Supplementary file 6 — Model statistics of neonatal mortality rate generated through Ljung. Box analysis of neonatal mortality rate in the 77 health deliveries. (DOCX 17 kb) [file 12884_2018_1708_MOESM6_ESM.docx]

**Additional File 6: Neonatal Mortality Rate Model Statistics**

|  | **Model statistics** | | | **Ljung-Box** | | |
| --- | --- | --- | --- | --- | --- | --- |
| **Model** | **Number of Predictors** | **Stationery R-squared** | **R-squared** | **Statistics** | **DF** | **Significance** |
| All 77 facilities | 3 | 0.11 | 0.11 | 17.34 | 17 | 0.43 |
| Urban based facilities | 3 | 0.18 | 0.18 | 14.19 | 17 | 0.65 |
| Rural based facilities | 3 | 0.09 | 0.09 | 13.70 | 17 | 0.30 |
| Maternity home | 3 | 0.18 | 0.18 | 19.54 | 17 | 0.65 |
| Level 4 health facilities | 3 | 0.21 | 0.21 | 21.89 | 17 | 0.19 |
| Level 5 health facilities | 3 | 0.08 | 0.08 | 33.29 | 17 | 0.10 |
| Level 6 health facility | 3 | 0.06 | 0.06 | 15.01 | 17 | 0.60 |
